# Interpretable machine learning for presurgical differentiation of Hürthle cell carcinoma and adenoma: a SHAP-augmented approach

**DOI:** 10.3389/fendo.2026.1865321

**Published:** 2026-07-20

**Authors:** Junyu Cao, Jing Li, Chuancheng Zhou, Jie Zhou, Kunxian Yang

**Affiliations:** Department of Breast and Thyroid Surgery, The First People’s Hospital of Yunnan Province, Kunming University of Science and Technology Affiliated Hospital, Kunming, Yunnan, China

**Keywords:** Hürthle cell neoplasm, machine learning, SHAP, thyroid cancer, XGBoost

## Abstract

**Background:**

The preoperative differentiation between benign Hürthle cell adenoma (HCA) and malignant Hürthle cell carcinoma (HCC) remains clinically challenging. This study aimed to develop an interpretable machine learning framework to improve diagnostic accuracy and assist clinical decision-making.

**Methods:**

We retrospectively enrolled 554 patients (280 HCA, 274 HCC) from a single center. Fifteen clinical, serological, and ultrasonographic variables were incorporated. Following rigorous feature selection via LASSO and Random Forest algorithms, four advanced machine learning models and logistic regression (LR) were trained and evaluated using 10-fold cross-validation. The SHapley Additive exPlanations (SHAP) and Individual Conditional Expectation (ICE) trajectories were utilized to interpret the optimal model globally and locally.

**Results:**

The XGBoost model demonstrated superior discriminative performance, achieving an Area Under the Curve (AUC) of 0.911, significantly outperforming LR (AUC = 0.746). Decision curve analysis confirmed its higher clinical net benefit. SHAP analysis demystified the algorithmic “black box,” identifying vascularity grade, absent halo sign, and elevated serum thyroglobulin as top malignant predictors. Furthermore, patient-specific ICE trajectories successfully simulated counterfactual clinical reasoning for misclassified cases.

**Conclusion:**

The SHAP-augmented XGBoost framework provides highly accurate, transparent, and personalized presurgical risk stratification for Hürthle cell neoplasms, potentially reducing unnecessary diagnostic thyroidectomies.

## 1 Introduction

Hürthle cell variants of thyroid neoplasms can be benign, as in Hürthle cell adenomas (HCA) or malignant, as in Hürthle cell carcinomas (HCC) (1, 2). The preoperative distinction between HCA and HCC continues to be a perplexing clinical diagnostic challenge (3). Fine-needle aspiration (FNA) biopsy, although recognized as the standard method for evaluating thyroid nodules, has limited diagnostic utility for Hürthle cell neoplasms due to its inability to assess capsular or vascular invasion (4). Hürthle cell neoplasms are primarily reported using the Bethesda system of reporting thyroid cytopathology, the coding and reporting of which indicates that the definitive diagnosis to ascertain a carcinoma can only be achieved by the analysis of a histopathological specimen showcasing capsular or vascular invasion, which cannot be made based on cytology alone (5). This perpetuates the scenario of conducting a malignancy exclusion, resulting in a high proportion of patients with benign adenomas being subjected to a diagnostic hemithyroidectomy or total thyroidectomy (6). This diagnostic surgical practice, despite being a prevalent approach in medicine, in addition to posing a high financial burden on a healthcare system, subjects the patients to numerous possible perioperative complications including damage to the recurrent laryngeal nerve and a permanent loss of parathyroid function (7–9).

The efforts directed at disproportional exclusion of malignancies in thyroid nodules have resulted in numerous attempts to identify reliable predictors to diagnose preoperatively (10). Several of these have been interpreted as different clinical demographics and serological markers (including thyroglobulin (Tg)) and some radiological characteristics (such as nodule size, calcification, and blood supply) in the case of thyroid nodules (11, 12). However, the majority of postulated classical statistical models in this context have been reliant primarily on simple logistic regressions that are predominantly limited by their direct linearity assumptions (13–15). Predictably, these have resulted in traditional statistical models which at best have generally been inadequate in capturing the non-linear interactions and threshold effects inherent in multidimensional clinical data, and have resulted in their statistical diagnostic performance and clinical relevancy to be less than optimal (16, 17).

Recently, many machine learning algorithms, especially ensemble learning algorithms like Extreme Gradient Boosting (XGBoost), and Random Forest, have shown notable ability in extracting useful information from various types of medical data (18, 19). Many of these algorithms have shown utility in challenging medical problems that traditional algorithms and even clinical and statistical methods have struggled with. However, despite their ability, these algorithms have yet to find their place in Clinical Decision Support Systems (CDSS) due to insufficient interpretation on how they arrive at decisions and predictions (20, 21). Many clinicians favor algorithms that yield lower diagnostic ability but offer better insight into how predictions are made, over algorithms with higher diagnostic ability but offering almost no insight into how predictions are made (22). Therefore, it is highly essential to offer machine learning algorithms that incorporate interpretability and high diagnostic ability.

In an attempt to fill this information gap, the current study will focus on formulating and rigorously testing a machine learning-based diagnostic model in preoperative distinction of HCC and HCA. It is important to mention that by taking a broad panel of 15 clinical, serological, and ultrasonographic indices, we aim to assess the output of various advanced algorithms. Furthermore, we utilized the SHapley Additive exPlanations (SHAP) framework and Individual Conditional Expectation (ICE) trajectories. The idea is aimed at demystifying the algorithmic decision process, which not only offers the importance of the global features but also offers counterfactual localized risk derivations on individual patients, enabling easy and personalized clinical management.

## 2 Materials and methods

### 2.1 Study design and patient selection

This single-center retrospective observational study was conducted at the First People’s Hospital of Yunnan Province. The study protocol was retrospectively approved by the Institutional Review Board (IRB) and Medical Ethics Committee of the First People’s Hospital of Yunnan Province (Approval number: 2026KHLL-0320). This approval was formally obtained prior to the commencement of data extraction and analysis. Due to its retrospective nature and the use of anonymized clinical data, the ethics committee waived the requirement for written informed consent. All procedures performed were in accordance with the ethical standards of the 1964 Declaration of Helsinki and its later amendments.

The study cohort consisted of patients who underwent diagnostic or therapeutic thyroidectomy at our institution between January 2014 and December 2023. The inclusion criteria consisted of the following: (1) patients with a definite post-operative histopathological diagnosis of a Hürthle cell neoplasm, namely a Hürthle cell adenoma (HCA) or Hürthle cell carcinoma (HCC), established by two independent pathologists following the World Health Organization criteria (necessitating unequivocal evidence of capsular and/or vascular invasion for HCC classification), which served as the definitive gold standard for benign versus malignant classification throughout this study.; (2) complete preoperative clinical, serological and high-resolution ultrasonographic data obtained within one month prior to surgery. The exclusion criteria included the following: (1) presence of concurrent non-Hürthle cell thyroid malignancies (e.g., papillary or medullary thyroid carcinoma); (2) history of a neck irradiation or thyroid surgery; (3) lack of medical record or the missing data on the 15 predictive variables of interest. Ultimately, there were 554 eligible patients (280 HCA and 274 HCC) enrolled in the analytical cohort.

### 2.2 Data collection and variable definition

Two researchers blinded to the final pathology results performed data extraction. A set of 15 preoperative features was systematically extracted from electronic medical records. The demographic and anthropometric variables were age (years), gender, and body mass index (BMI, calculated as weight in kilograms divided by the square of height in meters). Thyroid-stimulating hormone (TSH), free triiodothyronine (FT3), free thyroxine (FT4), serum thyroglobulin (Tg), thyroglobulin antibodies (TgAb), and thyroid peroxidase antibodies (TPOAb) were included as the serological assessments conducted before surgery.

Ultrasonographic assessments were retrospectively reviewed by two blinded, experienced sonographers using ultrasound systems equipped with 7–12 MHz high-frequency linear array transducers. Discrepancies in ultrasound feature interpretation were resolved by consensus with a third senior radiologist. The imaging characteristics that were extracted included the maximum size of nodules (in cm), the nodule volume (obtained through the ellipsoid formula V = length x width x depth x 0.52, in mL), presence or absence of a complete halo sign, and pattern of calcification (i.e., none, microcalcification [<1 mm], macrocalcification [>1 mm]), echotexture (homogeneous or heterogeneous), and vascularity grade (graded on a scale between Grade 0 and Grade 3 according to the Adler criteria using color Doppler flow imaging: Grade 0 indicates no internal blood flow; Grade 1 indicates 1–2 punctate or short rod-like flow signals; Grade 2 indicates 3–4 punctate flow signals or one longer vessel penetrating the lesion; Grade 3 indicates more than 4 punctate flow signals or an intricate reticular vascular pattern).

### 2.3 Data preprocessing and machine learning model construction

Prior to predictive modeling, the dataset was randomly partitioned into a training set (70%) and an independent test set (30%) using stratified sampling to preserve the original class distribution. To strictly prevent data leakage, all data preprocessing steps were executed after the data partitioning. The parameters for standardization (Z-score normalization) and the mappings for one-hot encoding of categorical variables were fitted exclusively on the training set. These identical scaling parameters and encoding rules were subsequently applied to transform the independent test set.

The selection of features and dimensionality reduction were primarily conducted using the Least Absolute Shrinkage and Selection Operator (LASSO) regression on the training set. The reduction of redundant coefficients to 0 was determined with the use of a ten-fold cross-validation process, which was applied to identify the feasible penalty parameter (λ). To rigorously cross-verify these linear selection outcomes, a Random Forest-based feature importance ranking and Recursive Feature Elimination (RFE) were applied in parallel. Ultimately, a consensus subset of 11 critical features (age, BMI, TSH, FT3, FT4, Tg, TgAb, TPOAb, nodule size, calcification, and vascularity grade) identified through this multidimensional evaluation was retained for subsequent model construction.

Then, four more sophisticated machine learning models, i.e., Extreme Gradient Boosting (XGBoost), Light Gradient Boosting Machine (LightGBM), Random Forest (RF) and Support Vector Machine (SVM), were constructed. A conventional Logistic regression (LR) model was made as a reference that is used as a baseline. We tested multiple hyperparameters by incorporating model-based hyperparameter optimization coupled with grid search, along with cross-validation in the training set to minimize overfitting while ensuring generalizability. All computational workflows were executed using R software (version 4.3.0) with a fixed random seed [set.seed(202401)] to ensure reproducibility. Specifically, the optimal hyperparameters for the XGBoost model (via the ‘xgboost’ and ‘caret’ packages) were determined within the following search space: maximum depth (3, 5, 7), learning rate (eta) [0.01, 0.05, 0.1], and number of trees (nrounds) [50, 100, 200]. The final optimal XGBoost configuration was set to max_depth = 4, eta = 0.05, and nrounds = 100.

### 2.4 Model evaluation and SHAP-based interpretability analysis

The performance metrics included the Area Under the Receiver Operating Characteristic (AUC-ROC) curve, the Area Under the Precision-Recall (AUPRC) curve, accuracy, sensitivity, specificity, Positive Predictive Value (PPV), Negative Predictive Value (NPV), and the F1-score. The 95% Confidence Intervals (CIs) for all metrics were calculated using 1,000 bootstrap resamples. Statistical comparisons of AUCs between models were performed utilizing DeLong’s test. Clinical utility was assessed via Decision Curve Analysis (DCA) by calculating the net benefit across a range of threshold probabilities. The smoothed calibration curves based on Generalized Additive Models (GAM) and the stability of the 10-fold cross-validation were also utilized as indicators of the models’ reliability.

In order to unravel the rational decision-making process of the optimal model, the SHapley Additive exPlanations (SHAP) model based on cooperative game theory was used. The SHAP summary plots, feature importance rankings, and dependence plots were used to visualize global interpretability elucidating the non-linear threshold effect of individual predictors. The demonstration included local interpretability with patient-specific waterfall plots of representative true positive, true negative, false positive, and false negative cases. Moreover, Individual Conditional Expectations (ICE) trajectories to particular misclassified patients were created to recreate counterfactual What-If clinical situations. All statistical tests and machine learning procedures were performed by means of R software (version 4.3.0). The normality of continuous variables was assessed using the Shapiro-Wilk test. Normally distributed variables were expressed as mean ± standard deviation and compared using Student’s t-test, whereas non-normally distributed variables were expressed as median [interquartile range] and compared using the Mann-Whitney U test. A P -value of less than 0.05 was termed statistically significant.

## 3 Results

### 3.1 Baseline characteristics and exploratory data analysis

**Figure 1:**
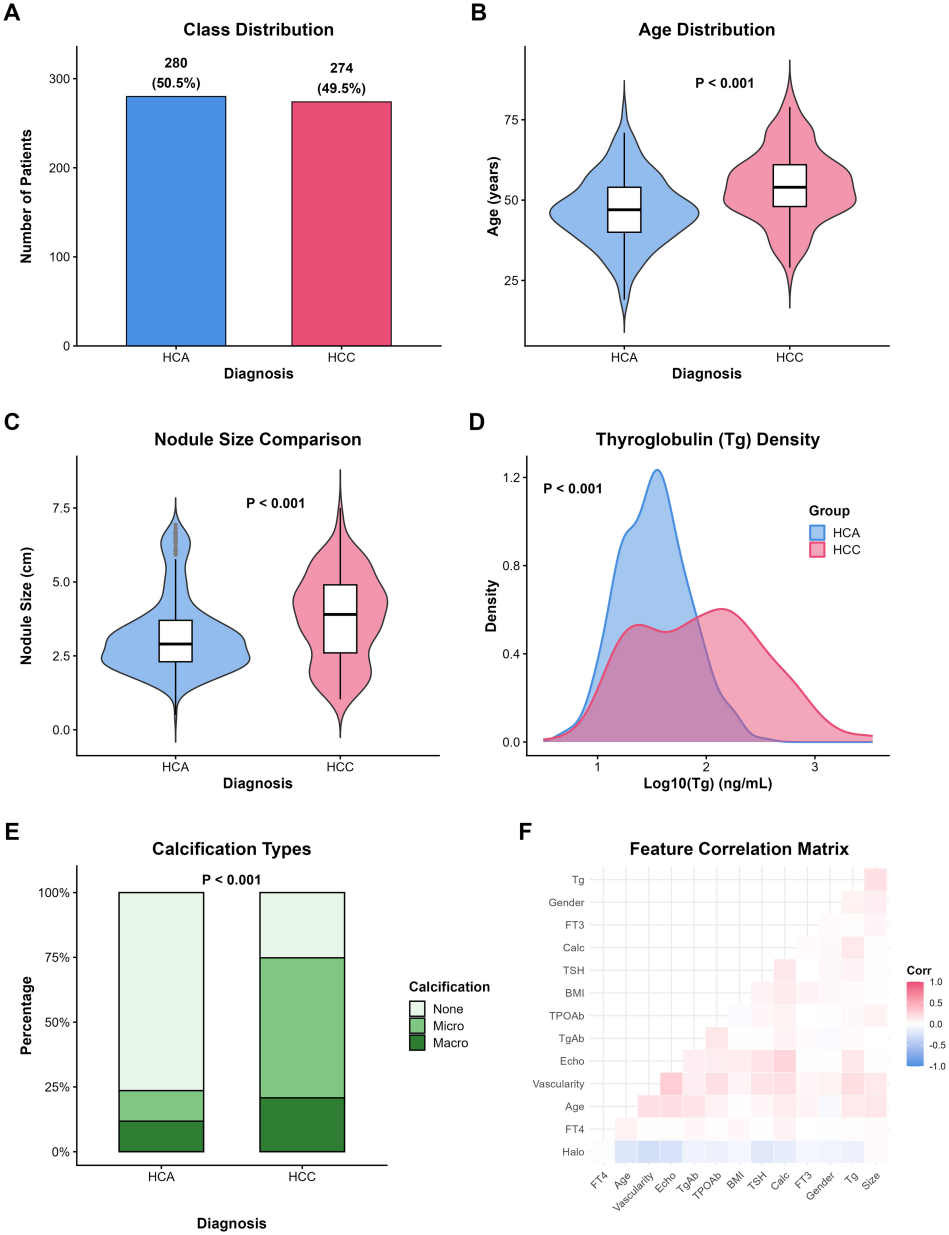
Exploratory data analysis and baseline distribution of key clinical and ultrasound features in patients with HCA and HCC. **(A)** Bar chart showing the balanced class distribution of the study cohort. **(B)** Violin and box plots comparing the age distribution between HCA and HCC groups. **(C)** Comparison of nodule size between the two groups. **(D)** Density plot illustrating the distribution of log-transformed Thyroglobulin (Tg) levels. **(E)** Stacked bar chart showing the proportion of different calcification types. **(F)** Pearson correlation matrix of the selected 15 features, demonstrating minimal multicollinearity among the variables. Statistical significance was determined using Student’s t-test or the Mann-Whitney U test for continuous variables, and the Chi-square test for categorical variables. HCA, Hürthle Cell Adenoma; HCC, Hürthle Cell Carcinoma.

A total of 554 patients with pathologically confirmed Hürthle cell neoplasms were included in this study, comprising 280 (50.5 percent) HCA cases and 274 (49.5 percent) HCC cases (**Figure 1A**). **Table 1** provides an overview of the baseline clinical, serological and ultrasonographic data of the cohort. Demographic analysis showed that patients in the HCC group were significantly older than those in the HCA group (mean age was 54.41 vs. 46.75 years, P < 0.001; **Figure 1B**). Body mass index was also significantly higher in the malignant cohort (P = 0.001), with no significant intergroup difference in gender (P = 0.198).

**Table 1:** Baseline Demographics, Serological and Ultrasound Characteristics of Patients with HCA and HCC.

Variables	Overall (N = 554)	HCA (n = 280)	HCC (n = 274)	*P*-value
Demographics				
Age (years)	50.54 ± 11.66	46.75 ± 10.75	54.41 ± 11.30	< 0.001
Gender, n (%)				0.198
Female	409 (73.8)	214 (76.4)	195 (71.2)	
Male	145 (26.2)	66 (23.6)	79 (28.8)	
BMI (kg/m ²)	23.75 ± 2.74	23.38 ± 2.67	24.13 ± 2.77	0.001
Serological Indices				
TSH (mIU/L)	2.05 [1.44–2.96]	1.85 [1.32–2.46]	2.27 [1.58–3.49]	< 0.001
FT3 (pmol/L)	4.85 ± 0.67	4.78 ± 0.55	4.92 ± 0.76	0.015
FT4 (pmol/L)	16.36 ± 2.24	16.13 ± 2.06	16.60 ± 2.39	0.015
Tg (ng/mL)	44.00 [22.38–110.12]	32.85 [18.87–53.87]	87.10 [28.34–229.27]	< 0.001
TgAb (IU/mL)	14.65 [7.73–32.58]	13.35 [7.50–27.33]	17.15 [8.90–40.08]	0.003
TPOAb (IU/mL)	14.30 [6.80–30.70]	11.45 [5.75–24.63]	18.70 [8.60–40.58]	< 0.001
Ultrasound Features				
Nodule Size (cm)	3.50 ± 1.42	3.21 ± 1.31	3.80 ± 1.46	< 0.001
Nodule Volume (mL)	8.95 [3.32–22.58]	6.10 [3.00–12.22]	15.35 [5.10–29.40]	< 0.001
Halo Sign, n (%)				< 0.001
Absent	245 (44.2)	52 (18.6)	193 (70.4)	
Present	309 (55.8)	228 (81.4)	81 (29.6)	
Calcification, n (%)				< 0.001
None	90 (16.2)	33 (11.8)	57 (20.8)	
Micro	181 (32.7)	33 (11.8)	148 (54.0)	
Macro	283 (51.1)	214 (76.4)	69 (25.2)	
Vascularity Grade, n (%)				< 0.001
Grade 0	74 (13.4)	62 (22.1)	12 (4.4)	
Grade 1	202 (36.5)	164 (58.6)	38 (13.9)	
Grade 2	184 (33.2)	40 (14.3)	144 (52.6)	
Grade 3	94 (17.0)	14 (5.0)	80 (29.2)	
Echo Texture, n (%)				< 0.001
Heterogeneous	284 (51.3)	76 (27.1)	208 (75.9)	
Homogeneous	270 (48.7)	204 (72.9)	66 (24.1)	

As to the ultrasonographic characteristics, the maximum diameters of the malignant nodules were significantly larger than those of the benign adenomas (3.80 vs. 3.21 cm, P < 0.001; **Figure 1C**). **Figure 1E** shows a fundamental difference in the distribution of calcification patterns in the two groups. Microcalcification was predominantly found in the HCC (54.0 percent) group, whereas the majority of HCA nodules (76.4 percent) had no calcification. Serological analyses revealed that preoperative thyroglobulin (Tg) levels were highly skewed and markedly elevated in patients with HCC (median 87.10 versus 32.85 ng/mL, P < 0.001; **Figure 1D**). Statistically significant differences between the groups also appeared in the rest of the thyroid function and antibody indices, such as TSH, FT3, FT4, TgAb, TPOAb, etc. (P < 0.05 in all cases; **Table 1**).

Before the development of a predictive model, a Pearson correlation matrix of the 15 selected clinical variables was created (**Figure 1F**). The heatmap showed that the continuous and categorical variables had little multicollinearity thus meeting the requirement of data structure prerequisite to the subsequent machine learning algorithms training.

### 3.2 Feature selection and dimensionality reduction

**Figure 2:**
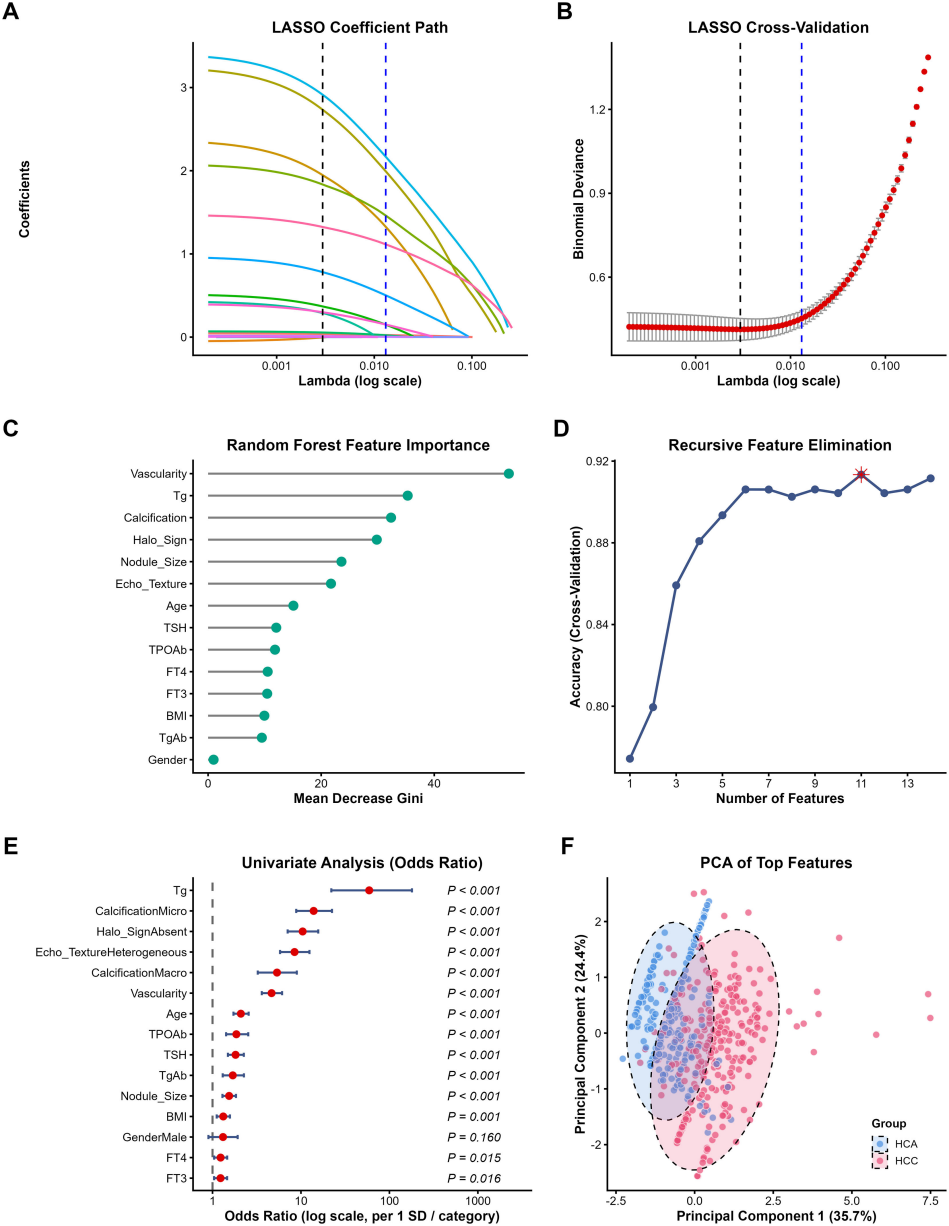
Feature selection and dimensionality reduction for the machine learning models. **(A)** LASSO regression coefficient path showing variables shrinking to zero as the penalty parameter (λ) increases. **(B)** Ten-fold cross-validation curve for the LASSO regression to determine the optimal tuning parameter (λ). **(C)** Feature importance ranking based on the Mean Decrease Gini derived from the Random Forest model. **(D)** Recursive Feature Elimination (RFE) curve illustrating the cross-validation accuracy across different sizes of feature subsets. **(E)** Forest plot displaying the standardized Odds Ratios (ORs) and 95% confidence intervals from univariate logistic regression analysis. **(F)** Principal Component Analysis (PCA) based on the top selected features, visualizing the spatial distribution and separability of the HCA and HCC clusters.

To construct a robust and clinically interpretable machine learning framework, it was imperative to prevent potential overfitting and redundancy caused by high-dimensional clinical and ultrasonographic data. As such, rigorous feature selection and dimensionality reduction was done utilizing a multi-algorithmic method. First, the least absolute shrinkage and selection operator (LASSO) regression was used to test the 15 candidate continuous and categorical variables. The coefficients of the irrelevant features were uniformly shrunk toward zero as the penalty parameter (i.e. λ) was increased as shown in the LASSO coefficient path (**Figure 2A**). The value corresponding to the minimum binomial deviance (log(λ) = 0.012) was used as the optimal tuning parameter in a ten-fold cross-validation process, which essentially reduced the number of redundant variables and identified an essential subset of predictors (**Figure 2B**).

In order to countercheck the linear selection outcomes with a non-linear influential approach, a Random Forest (RF) algorithm was used to evaluate the inherent feature importance. Based on the Mean Decrease Gini to assess the importance of each feature, vascularity grade was found to be the most critical one (Gini = 53.19), then came Serum Thyroglobulin (Tg) levels (Gini = 35.30), Patterns of calcification (Gini = 32.36), halo sign status (Gini = 29.83), and nodule size (Gini = 23.59) (**Figure 2C**). The high rank of these five characteristics in the RF model was greatly comparable with the variables that the LASSO penalty model kept. Moreover, the Recursive Feature Elimination (RFE) with cross-validation was done to determine the best model complexity. The performance curve showed that the accuracy initially increased sharply due to the use of an 11- feature subset which caused a stagnation in performance, after which additional variables did not yield significant improvements and could introduce noise (**Figure 2D**).

Univariate logistic regression along with algorithmic ranking of features were conducted to measure independent odds ratio (OR) of each variable to bridge the results of machine learning and conventional clinical epidemiology. In order to provide comparability among the measurements that would have different levels of measurement scales, highly skewed continuous variables were Z-score normalized before analysis. Elevated Tg levels (which were log10-transformed prior to standardization due to severe right-skewness, yielding a high apparent OR = 60.10 per 1-SD increase in the log scale), microcalcification (OR = 15.67), missing halo sign (OR = 12.45), and heterogeneous echo texture (OR = 4.32), which were presented in the forest plot (**Figure 2E**) were significantly related to the high risk of HCC (all P < 0.001). Lastly, the principal component analysis (PCA) was carried out using the highest ranking features to represent the multidimensional data structure. The two-dimensional scatter plot which explained 60.1% of the total variance (PC1 = 35.7, PC2 = 24.4) showed that there were distinct spatial clustering and reasonable separability of the HCA group and the HCC group (**Figure 2F**). All these multi-dimensional selection processes together made sure that only robust and clinically relevant predictors were retained to be used in predictive modelling later.

### 3.3 Construction and evaluation of machine learning models

**Figure 3:**
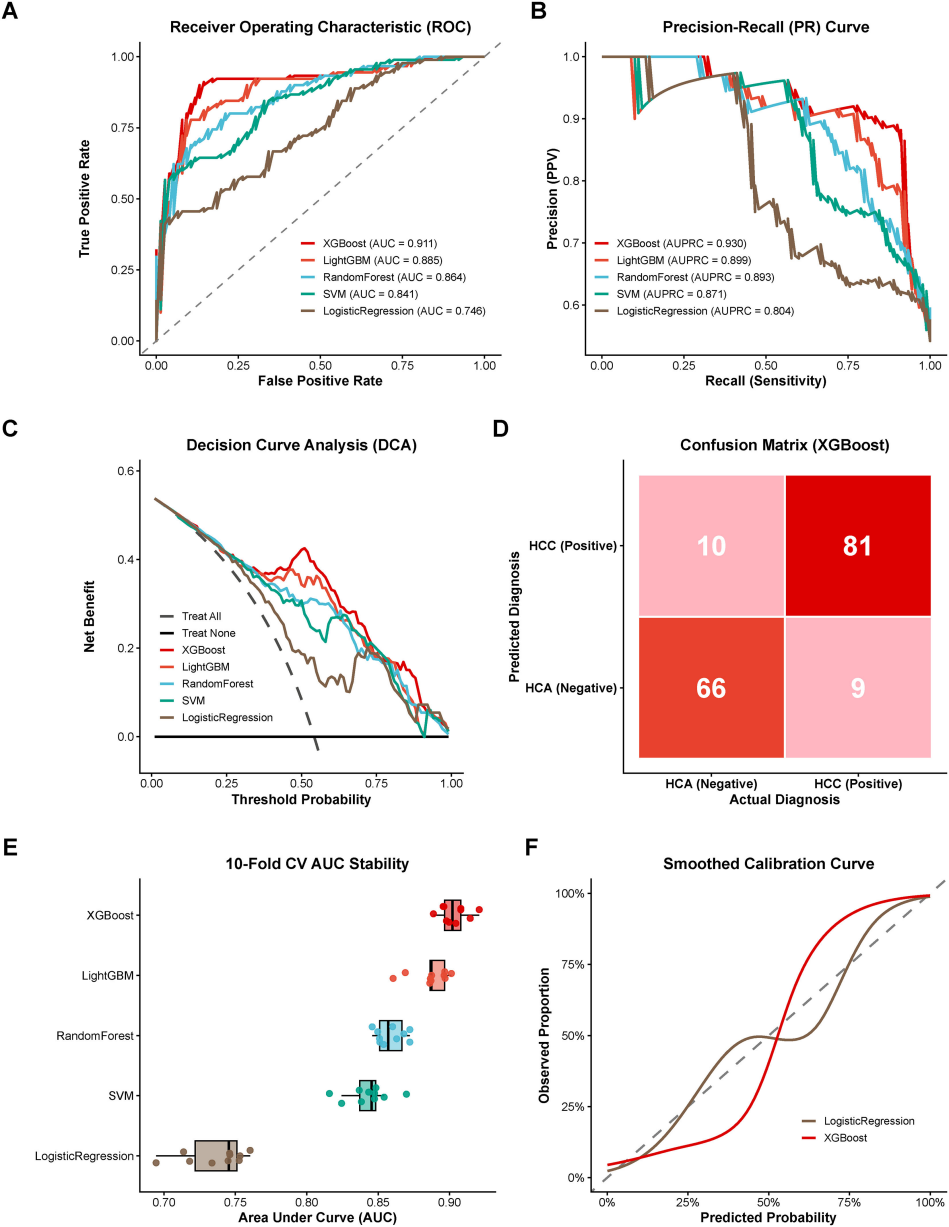
Performance evaluation and comparison of the five machine learning models. **(A)** Receiver Operating Characteristic (ROC) curves and corresponding Area Under the Curve (AUC) values. **(B)** Precision-Recall (PR) curves and AUPRC values. **(C)** Decision Curve Analysis (DCA) demonstrating the clinical net benefit of the models across various threshold probabilities. **(D)** Confusion matrix of the optimal XGBoost model, visualizing the accurate predictions and misclassifications. **(E)** Ten-fold cross-validation stability plot (boxplot with jittered points) showing the distribution and variance of AUC values across folds. **(F)** Smoothed calibration curves evaluating the agreement between predicted probabilities and actual observed proportions for the XGBoost and Logistic Regression models.

After the extensive feature selection, the refined predictor subset was leveraged to both train and test four state-of-the-art machine learning algorithms (XGBoost, LightGBM, Random Forest, and SVM) in addition to a conventional Logistic Regression technique. The capability of each model was appraised using both Receiver Operating Characteristic (ROC) and Precision-Recall (PR) curves evaluated on the independent test set. XGBoost, as illustrated in **Figure 3A**, demonstrated the best discriminative capability with Area Under the Curve (AUC) of 0.911, which is much higher than the traditional Logistic Regression model (AUC = 0.746). In line with the ROC analysis, the XGBoost has had the highest Area Under the Precision-Recall Curve (AUPRC = 0.930), which suggests that XGBoost can work with potentially unbalanced clinical cohorts better than other candidates (**Figure 3B**).

To determine the clinical utility of the models, we performed Decision Curve Analysis (DCA). As shown in **Figure 3C** (DCA curve), the XGBoost model achieved the highest net benefit across the majority of threshold probabilities among the evaluated models, indicating that, from a clinical perspective, interventions guided by this model would be more advantageous to patients than a treat-all or treat-none approach. Furthermore, the confusion matrix demonstrated that the XGBoost model accurately identified 81 cases of HCC and 66 cases of HCA, with an acceptable number of false positives (n = 10) and false negatives (n = 9) (**Figure 3D**).

A 10-fold cross-validation was performed to guarantee the robustness of the models and reduce the risk of overfitting. The stability plot (**Figure 3E**) depicted that XGBoost achieved a stable and higher performance (median AUC of about 0.90) and inter-fold variance was the lowest. Conversely, the Logistic Regression model was found to have a high degree of variation and lower overall accuracy when compared to the validation folds. Lastly, there was a strong level of consistency between the projected probabilities of the XGBoost model and the actual percentage of the HCC occurrence as observed in the calibration curve (smoothed) (**Figure 3F**) and not in the Logistic Regression model as a result, specifically, the mid risk ranges. Being superior in terms of the discriminative ability, stability, and clinical net benefit, XGBoost was chosen as the best model to be further subjected to interpretability analysis.

### 3.4 Global interpretability of the optimal XGboost model using SHAP

**Figure 4:**
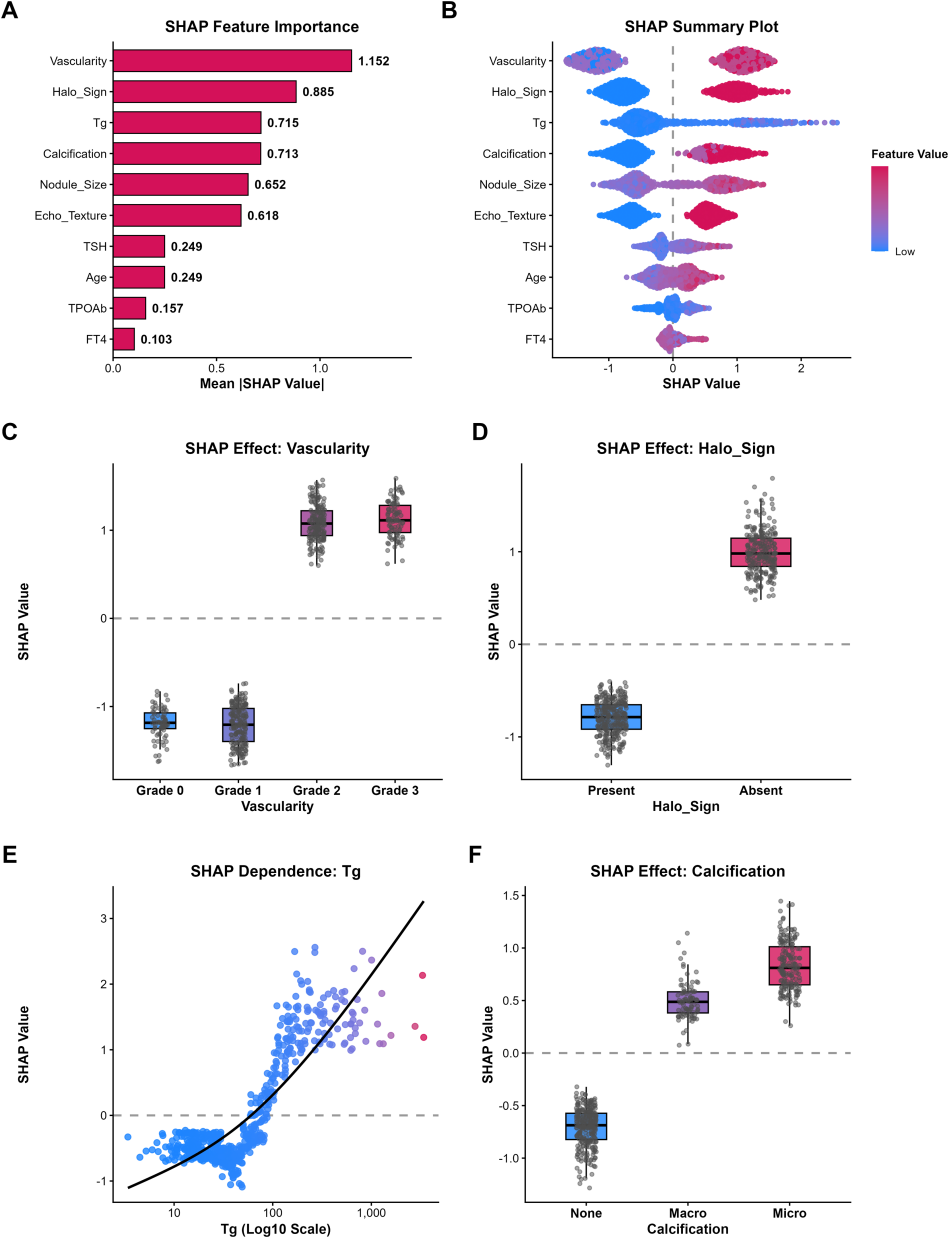
Global interpretability of the optimal XGBoost model using SHAP (SHapley Additive exPlanations) analysis. Panel **(A)** shows the global SHAP feature importance ranking based on the mean absolute SHAP values. Panel **(B)** is the SHAP summary plot illustrating the distribution of SHAP values for each feature**, where the** color gradient (blue to red) represents the actual feature values from low to high. Panels C through F display the SHAP dependence plots for the top four predictive features: Vascularity **(C)**, Halo Sign **(D)**, Thyroglobulin (Tg) mapped on a log10 scale **(E)**, and Calcification **(F)**. These plots demonstrate how specific feature values, whether categorical or continuous, non-linearly contribute to the model’s final prediction of malignancy.

To elucidate the decision-making logic of the optimal XGBoost model and mitigate the inherent “black-box” nature of ensemble learning algorithms, SHapley Additive exPlanations (SHAP) was employed for global interpretability analysis. With the help of the SHAP feature importance ranking (**Figure 4A**), the five most significantly influential predictors that defined the distinction between HCC and HCA were vascularity, lack of halo sign, serum thyroglobulin (Tg) level, calcification, and the size of the nodule. The ranking of importance based on Gini was rather stable to this ranking, which made this hierarchy transparent to the validity of the feature selection process.

A SHAP summary plot was created globally (**Figure 4B**) to plot the directional effect that all features have on the final prediction of the model. The color gradient of blue to red in this plot shows the value of the feature (low to high), whereas the horizontal line indicates the value of SHAP. The distribution clearly indicated that the positive value of SHAP was strongly correlated with higher values of vascularity, Tg, and calcification, thus generating more chances of malignancy being predicted. The data, on the other hand, shows that instances with low values of these features (blue dots) tended to affect the SHAP value negatively which suggests it had a benign inclination.

SHAP dependence plots were built to examine the non-linear aspect of the connection between the ranked features at the top and their risk of malignancy. The analysis of the vascularity results (**Figure 4C**) demonstrated the presence of a threshold effect; in particular, Grades 2 and 3 showed a significantly higher SHAP value, but Grade 0 and 1 were linked to the lower risk of developing HCC. The same categorical effect was also observed in the halo sign classification in which, the absence of a halo sign overwhelmingly pushed the prediction towards malignancy over the presence of a halo sign (**Figure 4D**). In addition, the dependence plot of Tg plotted on a log10 scale due to its strong skewed distribution (**Figure 4E**) was a monotonic, non-linear increment in SHAP values with an increase in Tg concentration beyond the range of approximately 100 ng/mL, with a plateau at the far ends. Lastly, it was found that the patterns of calcifications (**Figure 4F**) showed an increasing risk stratification, with microcalcification carrying the greatest risk of malignancy, followed by macrocalcification, while the absence of calcification served as a protective factor. These result indicate that the XGBoost model has been able to capture non-linear, complicated biological trends which are consistent with existing clinical and ultrasound-based guidelines.

### 3.5 Local interpretability and patient-specific “what-If” analysis

**Figure 5:**
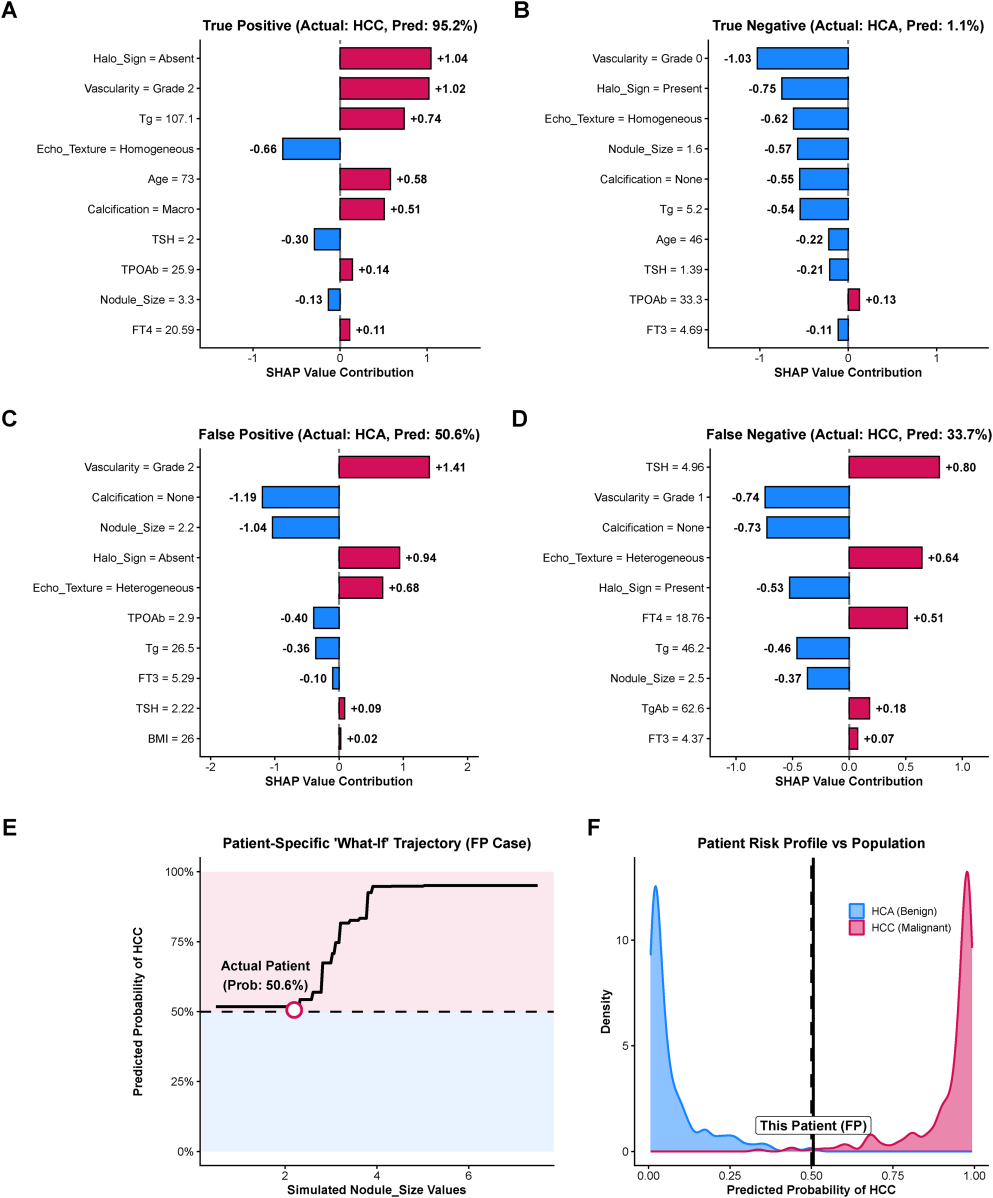
Local interpretability and patient-specific precision prediction analysis. **(A–D)** Local SHAP value contributions for four representative clinical cases: True Positive **(A)**, True Negative **(B)**, False Positive **(C)**, and False Negative **(D)**. Red bars indicate features pushing the prediction towards HCC (higher risk), while blue bars push towards HCA (lower risk). **(E)** Patient-specific “What-If” trajectory (Individual Conditional Expectation, ICE curve) for the False Positive case, simulating how the predicted probability of malignancy would shift if the nodule size varied continuously. **(F)** Density plot showing the predicted probability distribution of the entire cohort, with a vertical black line pinpointing the exact risk position of the specific False Positive patient.

While global SHAP analysis revealed the overarching decision patterns of the XGBoost model, local interpretability was essential to understand individual risk profiles and clinical decision-making. Consequently, four exemplary cases were taken out to illustrate the individual reasoning process of the model. In a typical instance of a true positive case (Actual: HCC, Predicted Probability: 95.2%), lack of a halo sign (+1.04 log-odds) and Grade 2 vascularity (+1.02 log-odds) served as the driving force that influenced the prediction toward malignancy, synergizing with high Tg and macrocalcification (**Figure 5A**). On the other hand, we had a large protective effect of the absence of vascularity (Grade 0, -1.03 log-odds) and the presence of a complete halo sign (-0.75 log-odds) on a true negative case (Actual: HCA, Predicted Probability: 1.1%), placing the prediction in the benign range (**Figure 5B**).

In order to critically assess the shortcomings of the model, cases that were misclassified were studied, in-depth. The presence of Grade 2 vascularity (+1.41) and the lack of a halo sign (+0.94) were the leading features that misled the model in a false positive situation (when a benign adenoma was misclassified as a malignant one) (**Figure 5C**). The absence of calcification (-1.19), and comparatively small nodule size of 2.2 cm (-1.04) however, had a strong negative effect but was not sufficiently strong to counter the accumulating malignant risk. On the same note, a false negative instance (Actual: HCC, Predicted Probability: 33.7) was incorrectly assigned a low risk score due to the deceptive presentation of Grade 1 vascularity (-0.74) and the lack of calcification (-0.73), which concealed the underlying malignancy risk, despite an increased level of TSH (+0.80) (**Figure 5D**).

In order to investigate how the borderline predictions could change when a dynamic change in the clinical appearances is done, an Individual Conditional Expectation (ICE) curve, or what are called What-If curve, was constructed on the above false positive case. When all other factors were constant and the nodule size was given a simulation of variations, the resulting trajectory demonstrated that the risk threshold was very non-linear (**Figure 5E**). At the patient’s actual nodule size of 2.2 cm, the projected probability was exactly at the 50.6 percent diagnostic boundary, which means that the patient was in the gray zone between the two ends of the population risk density profile (**Figure 5F**). It is worth noting that within the model’s mathematical framework, given the nodule had increased slightly further beyond 3.5 cm, the algorithm’s computed output of being malignant would have sharply risen to 100 percent. On the other hand, a somewhat smaller nodule size (< 2.0 cm) would have safely subclassified the patient to be benign. This individualized pathway matches the ability of this model to not just make arbitrary diagnostic scores, but also allow a simulated exploration of the algorithm’s decision boundaries, providing clinicians with dynamic reference points. However, it is crucial to note that ICE trajectories explain the model’s internal mathematical behavior based on observational data and should not be strictly interpreted as establishing direct biological causality.

## 4 Discussion

The preoperative differentiation between Hürthle cell carcinoma (HCC) and Hürthle cell adenoma (HCA) remains a formidable challenge in clinical endocrinology, primarily due to their overlapping cytological features in fine-needle aspiration biopsy (22). In this study, we constructed and tested an interpretable machine learning platform, showing that, when combined with SHAP analysis, the XGBoost algorithm is significantly more effective than conventional logistic regression in identifying HCC from HCA by incorporating complex, non-linear clinical and ultrasonographic predictors.

Our results are consistent with the most recent progress underlining the excellence of ensemble learning in comparison with the classical statistical models in high-dimensional medical data (23). Earlier research projects that tried to categorize indeterminate thyroid nodules have mostly used linear models or single-system ultrasonographic grading that failed to capture the complex relationships existing between physiological factors (24). In our cohort, XGBoost had an AUC of 0.911, representing a strong discriminatory ability without falling into the trap of overfitting based on analysis of the stability of the 10-fold cross-validation. Furthermore, the incorporation of Decision Curve Analysis (DCA) justified the fact that the higher diagnostic accuracy of XGBoost directly transfers into an increased net clinical benefit when compared to the traditional strategies. This measure is increasingly demanded by medical guidelines, because it independently evaluates the practical use of a predictive model beyond the statistical measures (25, 26).

One of the key contributions of this study is the ability to overcome the black-box dilemma inherent to advanced machine learning algorithms (27). Through the SHAP model, we clarified the fact that the most sensitive predictors of malignancy were vascularity grade, a halo sign absent, and high levels of serum thyroglobulin (Tg). These findings are biologically coherent with the defining histopathological criteria of HCC, namely capsular and vascular invasion. An absent halo sign on ultrasound typically reflects tumor disruption of the fibrous capsule, directly corresponding to capsular invasion. Similarly, hypervascularity is indicative of aggressive tumor angiogenesis, providing a physiological basis for vascular invasion. Furthermore, excessively elevated serum Tg levels reflect the destructive proliferation and functional hyperactivity of malignant Hürthle cells breaking through physiological barriers. Greater dependence on the SHAP plots indicated non-linear threshold patterns of risk. For instance, the risk contribution of Tg increased drastically once certain physiological limits were crossed, a phenomenon which logistic regression naturally assumes to be linear. Moreover, the localized What-If analysis of trajectory (ICE curve) provided the first-time understanding of the personal misclassification. We noted that borderline false-positive cases often exhibited contradictory clinical features, such as a small nodule size combined with an absent halo sign. Rather than dismissing these mistakes, the ICE curve demonstrated that a slight adjustment in one continuous variable can be very efficiently used to change the diagnostic threshold, enabling the clinician to have counterfactual interpretation, instead of a static diagnostic score.

While our findings are encouraging, some limitations to this work should be noted. First, the retrospective and single-centre nature of this study may lead to selection bias and limit the generalisability. Owing to the relative rarity of Hürthle cell neoplasms and the strict requirement for comprehensive 15-dimensional data, we were unable to perform external or temporal validation in the present study; thus, a potential risk of institutional overfitting remains. Furthermore, the diagnostic cut-offs and feature importance derived from our specific SHAP analysis might vary intrinsically when applied to different patient populations or when utilizing diverse ultrasound equipment and imaging protocols. Therefore, the models and thresholds established in our cohort must be rigorously validated through multicenter, prospective external cohorts across diverse demographic and clinical settings before they can be broadly applied in routine clinical practice. Second, even though we have included an extensive set of 15 serological and ultrasonographic indices in our model, we have not included complex molecular markers or genomic profiling, which could further improve the presurgical distinction of Hürthle cell neoplasms. The utilization of multi-omics data in future multicentric, prospective studies is justified to streamline this diagnostic framework before it can be utilized in clinical practice in its routine. Another important limitation concerns the interpretation of the machine learning explainability tools. It is imperative to emphasize that SHAP values and ICE trajectories elucidate the predictive behavior and internal mathematical mappings of the XGBoost model; they do not establish definitive biological causality. Furthermore, the ICE curve simulates changes in the prediction by varying a single feature while holding all other variables constant. In clinical reality, features such as nodule size, calcification, and vascularity are often physiologically correlated. Modifying one variable in isolation assumes feature independence and may generate synthetic, out-of-distribution data points that do not perfectly mirror true clinical evolution. Therefore, these interpretability visualizations should serve to augment, rather than replace, clinical judgment.

## 5 Conclusion

In summary, this paper created and tested an interpretable machine learning model and showed that the XGBoost algorithm significantly enhances the presurgical differentiation between Hürthle cell carcinoma (HCC) and Hürthle cell adenoma (HCA). The model was found to have a higher discriminative value and clinical net benefit than the traditional logistic regression by incorporating non-linear clinical, serological and ultrasonographic aspects. Moreover, the integration of SHAP analysis and Individual Conditional Expectation (ICE) trajectories effectively demystified the “black-box” nature of the algorithm providing clinicians with transparent, patient-specific counterfactual reasoning rather than static probabilistic scores. Although such results indicate that this advanced machine learning framework can be utilized to reduce the need for unnecessary diagnostic thyroidectomies, the outcomes should be interpreted with caution since the study is a retrospective, single-center design. Further prospective multicentric research involving the use of molecular profiling is needed to externally validate and optimize this diagnostic framework before its clinical use as an adjunctive decision support model.

## Data Availability

The raw data supporting the conclusions of this article will be made available by the authors, without undue reservation.
